# Multiple and Diverse *vsp* and *vlp* Sequences in *Borrelia miyamotoi*, a Hard Tick-Borne Zoonotic Pathogen

**DOI:** 10.1371/journal.pone.0146283

**Published:** 2016-01-19

**Authors:** Alan G. Barbour

**Affiliations:** 1 Department of Microbiology and Molecular Genetics, University of California Irvine, Irvine, California, United States of America; 2 Department of Medicine, University of California Irvine, Irvine, California, United States of America; University of Toledo School of Medicine, UNITED STATES

## Abstract

Based on chromosome sequences, the human pathogen *Borrelia miyamotoi* phylogenetically clusters with species that cause relapsing fever. But atypically for relapsing fever agents, *B*. *miyamotoi* is transmitted not by soft ticks but by hard ticks, which also are vectors of Lyme disease *Borrelia* species. To further assess the relationships of *B*. *miyamotoi* to species that cause relapsing fever, I investigated extrachromosomal sequences of a North American strain with specific attention on plasmid-borne *vsp* and *vlp* genes, which are the underpinnings of antigenic variation during relapsing fever. For a hybrid approach to achieve assemblies that spanned more than one of the paralogous *vsp* and *vlp* genes, a database of short-reads from next-generation sequencing was supplemented with long-reads obtained with real-time DNA sequencing from single polymerase molecules. This yielded three contigs of 31, 16, and 11 kb, which each contained multiple and diverse sequences that were homologous to *vsp* and *vlp* genes of the relapsing fever agent *B*. *hermsii*. Two plasmid fragments had coding sequences for plasmid partition proteins that differed from each other from paralogous proteins for the megaplasmid and a small plasmid of *B*. *miyamotoi*. One of 4 *vsp* genes, *vsp1*, was present at two loci, one of which was downstream of a candiate prokaryotic promoter. A limited RNA-seq analysis of a population growing in the blood of mice indicated that of the 4 different *vsp* genes *vsp1* was the one that was expressed. The findings indicate that *B*. *miyamotoi* has at least four types of plasmids, two or more of which bear *vsp* and *vlp* gene sequences that are as numerous and diverse as those of relapsing fever *Borrelia*. The database and insights from these findings provide a foundation for further investigations of the immune responses to this pathogen and of the capability of *B*. *miyamotoi* for antigenic variation.

## Introduction

*Borrelia miyamotoi* is a host-associated spirochete that is transmitted between its mammalian reservoirs by ticks of the genus *Ixodes* (reviewed in [1, 2]) Its vectors include *I*. *scapularis* in eastern North America, *I*. *pacificus* in far-western North America, *I*. *ricinus* in Europe, and *I*. *persulcatus* in Russia and Asia. These species are also the vectors of the Lyme disease agents of the genus *Borrelia*, as well as other zoonotic pathogens, like *Anaplasma phagocytophilum* and *Babesia microti*, in the same areas where *B*. *miyamotoi* is enzootic. In most regions in North America where *B*. *miyamotoi* and *B*. *burgdorferi* are sympatric, infection prevalence of *B*. *miyamotoi* in nymphal ticks is about one-tenth that for *B*. *burgdorferi* [1, 3], but in some areas, such as in northern California, the nymphal infection prevalences of the two *Borrelia* species are near-equal [4, 5]. While a North American strain of *B*. *miyamotoi* has been cultivated in the laboratory [6, 7], the growth rate is slow, and the yields of cultures are low. Consequently, infection of laboratory mice remains an important means for propagating infectious organisms in sufficient amounts for genomic studies and when plasmid loss is a concern [8].

*B*. *miyamotoi* was first identified in 1994 [9], but two decades passed before its recognition as a human disease agent, first in a case series from Russia [10] and then subsequently in reports from United States, Europe, and Japan [11–13]. Seroepidemiologic surveys indicate that the incidence of *B*. *miyamotoi* infection in the northeastern United States is similar to that for two other *I*. *scapularis*-transmitted diseases: anaplasmosis and babesiosis [14]. While *B*. *miyamotoi* infection does not appear to have the long-term sequelae that can occur with untreated Lyme disease [1], the acute infection sometimes resembles sepsis [10, 15] and has justified hospitalization, an infrequent consequence of acute *B*. *burgdorferi* infection. *B*. *miyamotoi*’s disposition for neuroinvasion was illustrated by cases of meningoencephaliltis in patients with pre-existing immunodeficiences [16, 17].

By phylogenetic criteria *B*. *miyamotoi* is more closely related to *Borrelia* species that cause relapsing fever, such as *B*. *hermsii* and *B*. *turicatae*, than it is to *B*. *burgdorferi* and other Lyme disease agents [9, 18, 19]. But in its biological traits *B*. *miyamotoi* differs from most other species in the relapsing fever clade in its preference for hard (or ixodid) ticks instead of soft (or argasid) ticks as its vector [20]. This exception in its life cycle leads to the question of whether *B*. *miyamotoi* is properly considered a relapsing fever agent. Clinical accounts of human *B*. *miyamotoi* infection include a minority of cases that had relapses of illness before antibiotic treatment was initiated [10, 15], but the natural history of untreated infection has yet to be well-defined in an experimental animal model. If *B*. *miyamotoi* in fact has the capacity for antigenic variation, which is a hallmark of relapsing fever [21], it plausibly is based on possession of a repertoire of polymorphic genes, the singular expression of which determines the immunodominant antigen of the cells.

As first documented for *B*. *hermsii* and subsequently for other relapsing fever species [22], a genomic schema that fits this specification is a plasmid-borne set of genes for either variable small proteins (Vsp) of ~20 kDa and variable large proteins (Vlp) of ~40 kDa [23]. On the basis of their amino acid sequences the family of Vlp proteins is further categorized into four sub-families or clusters—alpha, beta, gamma, and delta [24, 25]. Examples are the alpha sub-family protein VlpA7 (accession number P21876), the beta sub-family protein VlpB10 (P70905), the gamma sub-family protein VlpC5 (P70898), and delta sub-family protein VlpD17 (P32777). In *B*. *hermsii* and *B*. *turicatae* there is a duplicate copy of one of this set of polymorphic *vsp* and *vlp* genes [26, 27]. Only this duplicate copy is transcribed at a single expression site [28], thereby providing the antigenic identity to the cell. As might be predicted if evasion of the immune system was a pathogen’s strategy, diversity among the Vsp and Vlp proteins of a given lineage is substantial [25, 29].

My objective was to search for and characterize *vsp* and *vlp* genes in the reference North American strain of *B*. *miyamotoi*. Sequences homologous to *vsp* genes had been identified in *B*. *miyamotoi* strains from Japan [30] and North America [19]. But these studies were limited in scope, and the broader organization of these sequences was not revealed. Finding a variable gene repertoire of a scale possessed by *B*. *hermsii* was not assured, though. *B*. *anserina*, another species in the relapsing fever agent clade [19], appears to have no *vlp* genes and only a single *vsp* (accession number KJ136518), on the basis of publicly-available DNA sequences (BioProject 195596) of its genome (unpublished finding). Moreover, while identification of the variable regions of individual *vsp* and *vlp* sequences is achieveable with high-coverage, short-read sequencing, the duplicated conserved sequences at their 5’ and 3’ ends makes unambiguous assembly of longer contigs with full-length genes a challenge [25]. In addition, the genetic distance of *B*. *miyamotoi* from other species precluded a resequencing procedure in which short reads were mapped to a reference. These considerations led to a hybrid approach with supplementation of the previously-acquired database of short-reads with the longer reads obtainable with real-time DNA sequencing from single polymerase molecules [31]. This allowed not only identification of several complete or near-complete *vsp* and *vlp* sequences but also greater stretches of the plasmids bearing them.

## Materials and Methods

### Ethics statement

All animal work was conducted with approval of University of California Irvine’s Institutional Animal Care and Use Committee (protocol 2080–1999). Mice were housed under ABSL2 containment in an Association for Assessment and Accreditation of Laboratory Animal Care-approved facility. This study was carried out in strict accordance with the recommendations in the Guide for Care and Use of Laboratory Animals of the National Institutes of Health.

### Bacterial strain and propagation

Strain LB-2001, which was isolated from *I*. *scapularis* ticks collected in Connecticut in 2001 [18], was used. The isolate was provided to the author by Michele Papero and Durland Fish of Yale University in 2003 and was kept frozen in individual aligquots at -80°C. It had been passed three times in mice, and never under in vitro conditions, before further expansion for this study in adult CB17 severe-combined immunodeficient (SCID) mice from Charles River Laboratories (Wilmington, MA) as described [32]. Infections of the mice were monitored by phase-microscopy of tail vein blood as described [25]. When bacterial densities in blood reached 0.5–1.0 x 10^7^ cells per milliliter, mice were terminally exsanguinated under anesthesia; blood was collected in heparinized tubes. The isolate was registered as BioSample SAMN02604147 as part of BioProject PRJNA19262 (http://www.ncbi.nlm.nih.gov).

### Short-read sequences

For our previous study of the chromosome of *B*. *miyamotoi* LB-2001, we obtained ~1.7 million Ion Torrent (Life Technologies, Carlsbad, CA) reads of 50–250 nucleotides from a total DNA extract of whole blood of *Mus musculus* mice infected with this strain [19, 32]. For the present study, I examined reads that did not map to the LB-2001 chromosome under moderately stringent conditions. The 1,135,420 unmapped reads, which were presumed to be mainly extrachromosomal DNA of *B*. *miyamotoi* as well as some residual mouse DNA, were used for a de novo assembly using the Assembly Cell algorithm of CLC Genomics Workbench v. 8.1 (Qiagen, Valencia, CA). The resultant assembly had 101 contigs of ≥2000 nt, for a total of 392,484 nt from 256,835 reads. These contigs ranged in size from 2016 to 29,162 nt, the average size was 3886 nt, and the average coverage was 99X.

### Long-read sequencing and assembly

For this experiment another harvest of strain LB-2001 was again produced by propagating in SCID mice as described [19, 32]. Total DNA was extracted from heparinzied whole blood with Qiagen’s DNeasy Blood/Tissue Kit and then treated with RNase I. Library preparation and sequencing using the Single Molecule, Real-Time (SMRT) DNA approach on a PacBio RS I instrument (Pacific Biosciences, Menlo Park, CA) was performed in University of California Irvine’s Genomics High-Throughput Facility (http://ghtf.biochem.uci.edu). In brief, genomic DNA was purified with Genomic Tip 500/G columns (Qiagen) and was sheared using the g-Tube (Covaris, Woburn, MA), following the Pacific Biosciences protocol for low-input (10 kb) preparation and sequencing. Agencourt AMPure magnetic beads (Beckman Coulter, Brea, CA) were used to remove salt according to the PacBio template and preparation sequencing instructions. A SMRTbell Template Preparation kit (Pacific Biosciences) was used to construct a 3–10 kb library, and the size was determined using a 2100 Bioanalyzer (Agilent, Santa Clara, CA). The Pacific Biosciences calculator (v. 2.0) was used to determine the amount of primer and polymerase (DNA/Polymerase binding kit 2.0), and samples were sequenced with the MagBead Seq v1 protocol (Pacific Biosciences). Default filters removed reads of less than 50 nt and 0.75 accuracy.

Two SMRT cells yielded a total of 250,161 one-pass reads of 50 to 22,451 nt for a total of 540,499,213 bases and average length of 2237 nt. Error correction of the long PacBio reads with the total Ion Torrent reads was carried out with the default parameter settings of the pacBioToCA module of the Celera Assembler v. 8.1, and the output was provided as fastq and fasta files [33]. These were imported for analysis by the suite of programs of CLC Genomics Workbench v. 8 (Qiagen) and for the database of a local stand-alone WWW Blast Server v. 2.2 [34]. Thirty-eight percent of the reads mapped to a *Mus musculus* genome, but for all mouse chromosomes except the mitochondrial the coverage was <0.05X. Using the extrachromosomal de novo contigs as the reference, the coverage for putative plasmid sequences ranged between 5X and 10X.

De novo Ion Torrent contigs of ≥500 nt and the corrected PacBio reads with possible *vsp* and *vlp* genes were identified with TBLASTN searches of de novo Ion Torrent contigs and error-corrected PacBio reads with selected Vsp and Vlp protein sequences of *B*. *hermsii* strain HS1 and Vsp1 of *B*. *miyamotoi* LB-2001. Candidate *vsp* or *vlp*-bearing sequences were used in turn for BLASTN searches on the local blast server to identify PacBio reads that overlapped the probe sequence by >200 bases with neither mismatches nor gaps. The resultant set of overlapping reads was then edited to produce a single continuous sequence.

### Accession numbers

GenBank/EMBL/DDBJ accession numbers for the large fragments of plasmids lpB, lpD, and lpE are CP010328, KR869094, and KU041636, respectively. The coding sequences for plasmid replication and partition proteins PF-32, PF-49, and PF-50 of the megaplasmid of *B*. *miyamotoi* LB-2001 are given in accession number KU145468. The 6035 bp of the sequence of the small plasmid cpD of *B*. *miyamotoi* LB-2001 has accession number KT355574.

### Other sequences

These included FR64b chromosome (CP004217) and its plasmid fragments (GenBank assembly number GCA_000568695), specifically, plasmid fragments 17 (CP004234), 36 (CP004253), and 39 (CP004256), and *B*. *hermsii* strain HS1 coding sequences for Vsp6 (DQ166207), Vsp22 (EF156411), and Vsp24 (EF187445), a *B*. *hermsii* strain YBT *vsp* gene (CP005724), *B*. *turicatae* strain Oz1 coding sequences for VspA (AF129434) and VspB (AF049852), and coding sequence for *B*. *miyamotoi* LB-2001 *flaB* gene (AGT27144). The following ParA-type proteins of strain B31 of *B*. *burgdorferi* were used: BBA20 (AAC66247), BBB12 (AAC66318), and BBN32 (AAF07673). The 2.6 Gb *Mus musculus* sequence of Genome Reference Consortium Mouse Build 38 (GCA_000001635.2) served as the mouse reference. The Shuffle DNA algorithm (www.bioinformatics.org/sms2/shuffle_dna.html) was used to randomly mix the order of bases of a sequence while retaining the original base composition.

### Sequence analysis

Open reading frames (ORFs) of ≥150 bp with any codon as a start were identified and searched against the National Center for Biotechnology Information (NCBI) nonredundant protein database using BLASTP [34]. The BLAST search criteria for considering an ORF as homologous to a deduced protein of an another organism were the following: *E* values of <10^−5^, pairwise amino acid identities of >40%, and >50% coverage of the smaller protein. Further annotation took into account relationships with proteins whose functions have been identified or which were commonly known in the literature.

The convention for naming *vsp* and *vlp* genes and their deduced proteins was to arbitrarily assign a numeric suffix to a *vsp* or *vlp* ORF, e.g. *vsp1*, according to their position in a contig, proceeding from left to right, and then moving to the next contig once all the putative *vsp* and *vlp* sequences of one contig had been designated. The *vlp* coding sequences, either complete or partial at the 5’ end, were further differentiated by sub-family membership, e.g. “*vlpA1*”, “*vlpC2*”, and “*vlpD6*” in the case of alpha, gamma, and delta sub-families. Some other ORFs were assigned designations that indicated their homology with specific proteins or a paralogous family (PF) protein of *B*. *burgdorferi* strain B31 [35], the source for the first genome sequence in the genus *Borrelia* [36].

Alignment of DNA and protein sequences was carried out with ClustalX v. 2 [37], and were codon-aligned manually with MacClade v. 4.10 (Sinauer Associates, Inc., Sunderland, MA). Distance-based clustering algorithms for ungapped alignments of proteins were neighbor joining with the BioNJ protocol and Poisson distances. Phylogenetic inference was carried out by maximum likelihood estimation, as implemented by PhyML v. 3.0 (Guindon et al., 2010) in the SeaView suite version 4.5.4 [38]. SplitsTree4 (http://www.splitstree.org) was used for distance-based clustering of ungapped nucleotide alignments and computation of an unrooted phylogenetic network [39].

### RNA-seq

Total RNA was isolated from ~1 ml of heparinized whole blood from each of 3 infected male SCID mice using a RNeasy Mini Kit (Qiagen, Valencia, CA). There were ~5 x 10^6^ spirochetes per milliliter of blood at the time of collection. After RNA was eluted from the columns in RNase-free water, residual DNA was removed by treatment with DNase I (Thermo Fischer Scientific) during further processing with a RNA Clean & Concentrator-5 kit (Zymo Research). The sample was depleted of ribosomal RNA by incubation with the beads of Ribo-Zero Magnetic Kit for Gram-positive bacteria (Epicentre, Madison, WI) followed by ethanol precipitation. Synthesis of cDNA with random hexamer primers was carried out with a Maxima First Strand cDNA Synthesis Kit (Thermo Fischer Scientific). The cDNA was then treated with RNase I before processing with a DNA Clean & Contrator-5 kit (Zymo Research). The resultant cDNA was sheared enzymatically, and adapters were ligated with an Ion Express Plus Fragment Library Kit (Life Technologies, Carlsbad, CA). The products were size-selected with the E-Gel electrophoresis system (Life Technologies), attached to Ion Sphere Particles using an OneTouch 200 Template kit (Life Technologies), subjected to emulsion PCR on an Ion Torrent OneTouch apparatus, and then sequenced on an Ion Torrent Personal Genome Machine with Ion 316 chips and Ion PGM 200 Sequencing Kit (Life Technologies). The RNA-Seq program in the CLC Genomics Workbench, version 8.1 (Qiagen) was used to enumerate reads mapping to reference sequences with these criteria: minimum length fraction of 0.5, minimum similarity fraction of 0.9, and costs of 2, 3, and 3 (out of 3) for mismatches, insertions, and deletions, respectively.

## Results and Discussion

### Three plasmid fragments bearing *vsp* and *vlp* sequences

The assembled contigs of extrachromosomal DNA included one of 37.5 kb, which contained the *nrdEFI* and *thyX* genes (KJ141201) of the 150–180 kb megaplasmids of relapsing fever *Borrelia* species [40], and another of 29 kb, which contained the plasmid partition proteins of the megaplasmids [41, 42]. There was also a 6 kb plasmid (accession number KT355574), which corresponded to small plasmids that are found in relapsing fever species but not Lyme disease species (unpublished findings). Neither the megaplasmid sequences nor the small plasmid sequence contained a discernible *vsp* or *vlp* gene sequence.

**Fig 1:**
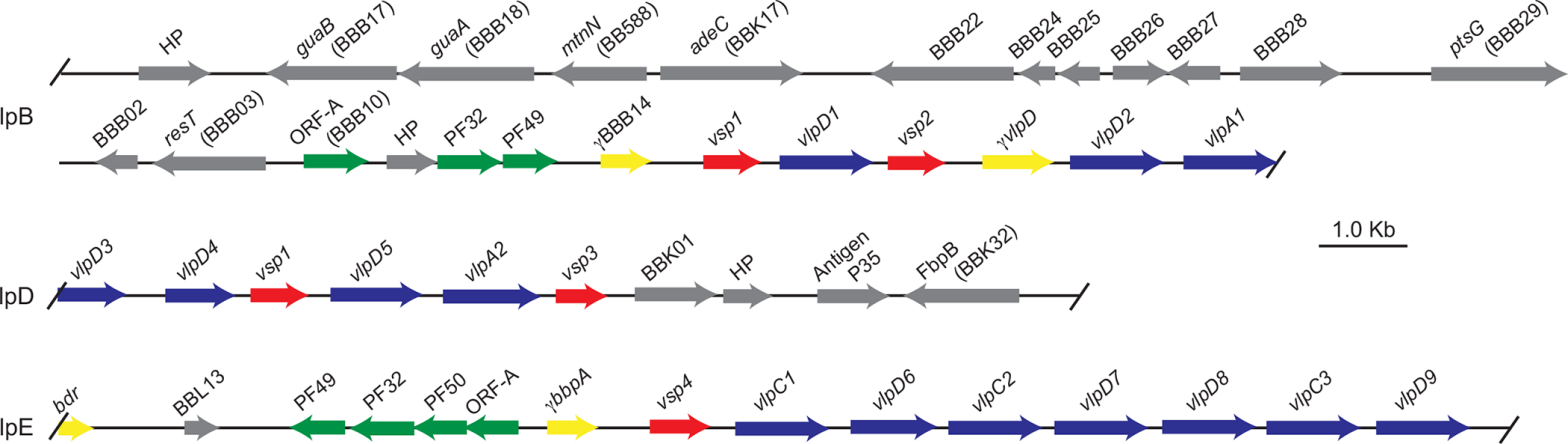
Physical maps of three fragments of strain LB-2001 *Borrelia miyamotoi* plasmids lpB, lpD, and lpE that contain sequences for *vsp* genes (red arrows), *vlp* genes (blue arrows), and/or plasmid partition and replication genes (green arrows). Also shown are the locations of other open reading frames (gray arrows), which are indicated by gene names (e.g. *guaA* and *bdr*) or by *Borrelia burgdorferi* open reading frames names (e.g. BBB22) or paralogous family (PF) numbers (e.g. PF50) [35, 36]. When an ORF had no discernible homology with a protein in the GenBank database, it was designated a hypothetical protein (HP). The *vsp* and *vlp* genes are further distinguished by an arbitrarily assigned number (e.g. *vsp2*) and, in the case of *vlp* genes, by appending their membership in a *vlp* subfamilies alpha, gamma, and delta with a “A”, “C”, or “D”, respectively. Pseudo genes are indicated by a “*Ψ*” prefix and yellow arrows. The arrowheads indicate the direction of transcription. The start and stop positions for each open reading frame, the protein translations, and additional annotation are given in the GenBank/EMBL/DDBJ deposits CP010328 (lpB), KR869o94 (lpD), and KU041636 (lpE), respectively. The scale in kilobases (kb) is indicated by the size marker.

**Fig 2:**
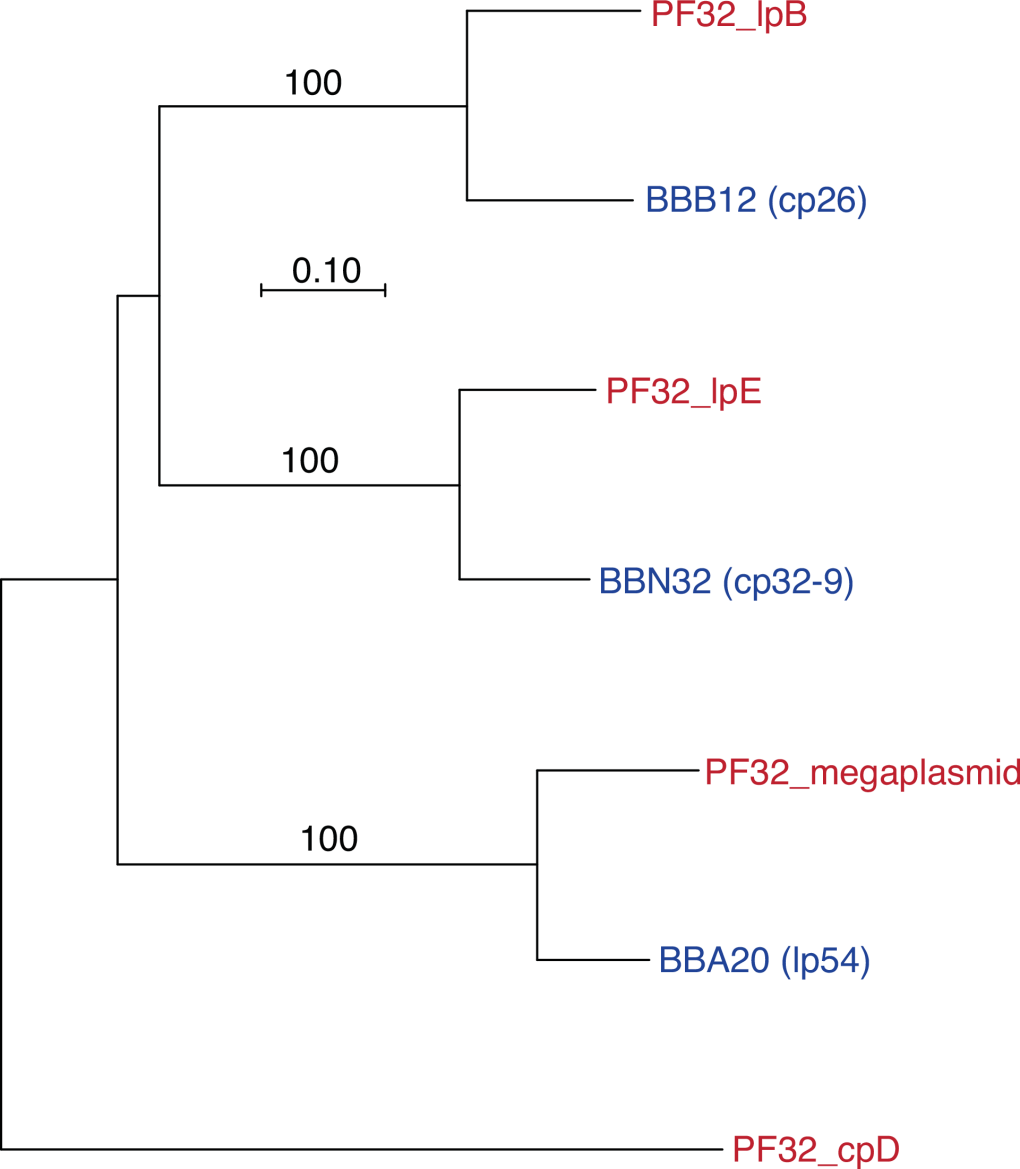
Phylogram of amino acid sequences for four PF32 (ParA) paralogs (red text) of strain LB-2001 *B*. *miyamotoi* and corresponding PF32 sequences and their ORF designations (blue text) of strain B31 *B*. *burgdorferi*. The plasmid fragment origin of the PF32 sequence of *B*. *miyamotoi* is indicated. The associated plasmids for each of *B*. *burgdorferi* sequences are given parentheses. Nodes with bootstrap values of >70% support by neighbor-joining distance criteria from 1000 replicates are indicated. The scale bar represents nucleotide substitutions per site.

As detailed below, iterative TBLASTN and BLASTP searches revealed three contigs of >10 kb that contained coding sequences homologous to *vsp* and *vlp* genes of other *Borrelia* species. These plasmid fragments were 30,673 bp, 11,152 bp, and 16,042 bp in length and were designated lpB, lpD, and lpE, respectively (Fig 1). The lpB and lpE contigs had ORFs that were orthologous to plasmid replication and partition proteins of plasmids of the genus *Borrelia*. These included coding sequences for PF32 or ParA proteins, PF49 proteins, and PF50 proteins, as well as “ORF-A” coding sequences that were commonly found in association with genes for partitioning proteins of plasmids [35, 42]. The aligned ParA proteins of lpB and lpE were only 48% identical over their lengths. Fig 2 is a phylogram of the PF32 proteins of the megaplasmid, lpB, lpE, and the small plasmid cpD, as well as orthologous sequences of *B*. *burgdorferi*. The lpB ParA protein’s ortholog in *B*. *burgdorferi*, BBB12, is found on the cp26 plasmid, while lpE’s PF32 protein is more similar to those, such as BBN32, of the cp32 plasmids of *B*. *burgdorferi*. The *B*. *miyamotoi* protein found on the megaplasmid fragment is most similar to the PF32 protein BBA20 of the lp54-type (“A”) plasmids [35, 42]. By BLASTP and TBLASTN searches, there was no detectable ortholog of the cpD ParA protein among the published genomes of Lyme disease group *Borrelia* species.

The observed diversity of four PF32/ParA proteins and their correspondences to distinctly different replicons in *B*. *burgdorferi* indicate the presence of at least four different types of plasmids in *B*. *miyamotoi*. This is consistent with Hamase et al.’s report of 7–9 plasmids in strains of *B*. *miyamotoi* from *I*. *persulcatus* ticks in Japan [30]. The lpD contig did not include ORFs with a discernible replication or partition function, so a separate plasmid status cannot be inferred. But two of its ORFs for hypothetical proteins were homologous to proteins of the lp36 (or “K”) class of plasmids of Lyme disease *Borrelia* spp. [43].

### The lpB fragment is largely homologous to the cp26 plasmid of *B*. *burgdorferi*

A shared ancestry between the 31 kb lpB sequence and the 26 kb cp26 circular plasmids of Lyme disease *Borrelia* species was further documented by discovery on lpB of coding sequences for the following proteins (with gene name) that typify cp26-type (denoted by “B” in ORF name) plasmids [44] (Fig 1): inosine 5-monophosphate dehydrogenase (*guaB*), GMP synthase (*guaA*), uracil-xanthine permease (BBB22), PTS glucose transporter subunit IIB (*ptsG*), and, what has been essential for other *Borrelia* spp., the telomere resolvase (*resT*) [45]. Other ORFs were orthologous to cp26 genes BBB24 through BBB28 [35]. The exceptions to the overall similarity between lpB and cp26 replicons were coding sequences for an adenine deaminase (*adeC*), which is encoded by BBK17 of the lp36 plasmid of *B*. *burgdorferi*, and for a 5'-methyladenosine nucleosidase (*mtnN*), which is encoded in *B*. *burgdorferi* by the chromosomal ORF BB588.

In *B*. *hermsii* the replicon bearing a cp26-like plasmid partition gene (CP002636) is a 53 kb linear plasmid [46]. The difference in the order of the genes between the lpB fragment and cp26, e.g. a BBB02-like ORF follows the BBB29-like ORF on lpB, may be attributable to retention in the lineage of *B*. *miyamotoi* and other relapsing fever species of a linear structure for a plasmid whose counterpart in the divergent lineage of *B*. *burgdorferi* and other Lyme disease species is circular, as suggested by Chaconas [47]. I found no PacBio reads that spanned the 5’ and 3’ ends of the lpB fragment, which would be an expected finding if lpB had a circular topology. However, since comparatively little is known about the telomeric sequences of the linear plasmids of relapsing fever *Borrelia* species, it was not possible to assign definite termini on the basis of sequence.

### *Vsp* and *Vlp* genes

Hamase et al. reported that multiple restriction fragments of the Asian strain HT31 of *B*. *miyamotoi* hybridized with a probe incorporating a *vsp* sequence they had identified [30]. These findings were consistent with the presence of more than one *vsp* in the genome. Subsequently, I identified a *vsp* gene in strain LB-2001 and designated this *vsp1* (KF031441) [19]. Further sequencing placed this sequence with its particular 5’ flanking region on the 31 kb lpB fragment (Fig 1) and revealed another Vsp coding sequence, *vsp2*, and four Vlp coding sequences—*vlpD1*, *vlpD2*, *vlpA1*, as well as a pseudogene of a delta sub-family *vlp*—downstream of and in the same orientation as *vsp1*.

The fragments of plasmids lpD and lpE had additional sequences for different *vsp*, *vlpA*, and *vlpD* alleles. Fragment lpE contained coding sequences for three different gamma sub-family Vlp proteins—*vlpC1*, *vlpC2*, and *vlpC3*—besides Vsp and delta sub-family VlpD proteins. No beta sub-family sequences were identified by TBLASTN with VlpB9, VlpB10, VlpB12, and VlpB14 sequences of *B*. *hermsii* [29] in either the de novo assemblies or the PacBio reads. This is in contrast to *B*. *turicatae*, *B*. *parkeri*, *B*. *persica*, *B*. *coriaceae*, *B*. *hispanica*, and *B*. *duttonii*, all of which have beta sub-family Vlp genes, as exemplified by the protein sequences with accession numbers AF130429, AHH10139, WP_051374011, AHH11256, WP_038359110, and ACH94076, respectively.

Six of the 19 *vsp* and *vlp* sequences of the lpB, lpD, and lpE plasmids would encode a full signal peptide for these lipoproteins. This proportion is similar to what was found for the silent *vsp* and *vlp* sequences in the *B*. *hermsii* genome [25]. Another 10 of the *B*. *miyamotoi vsp* and *vlp* genes had at least part the coding sequence for peptide sequence, including a consensus four amino acid site for the signal peptidase for lipoprotein processing. The remaining three ORFs began within a few residues of where the signal peptidase site would be expected. Adjacent to these *vsp* and *vlp* sequences there was not disernibly a repetitive sequence that was analogous to the Downstream Homology Sequence of *B*. *hermsii* [25].

**Fig 3:**
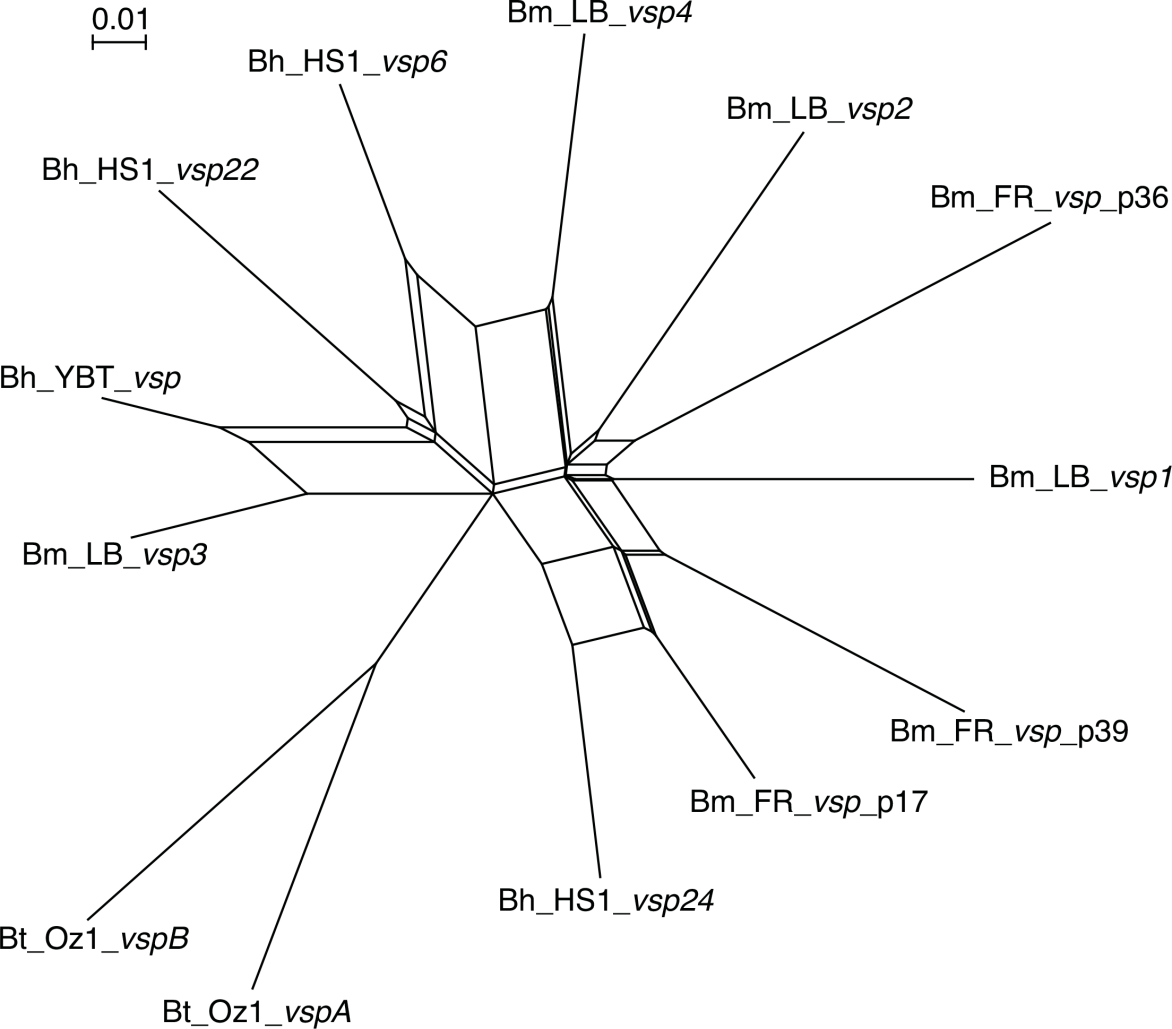
Unrooted distance phylogram with recombination network for codon-aligned partial *vsp* genes of selected *Borrelia* species, as implemented by the SplitsTree algorithm. The sequences were from strain LB-2001 of *B*. *miyamotoi* (Bm_LB) from North America, strain FR64 of *B*. *miyamotoi* (Bm_FR), strains HS1 and YBT of *B*. *hermsii* (Bh_HS1 and Bh_YBT), and strain Oz1 of *B*. *turicatae* (Bt_Oz1). The 3 *vsp* sequences of FR64b were further distinguished by the plasmid fragment contig (e.g. “p36” for “plasmid fragment 36”) they were located on. The sequences in NEXUS format for the alignment are provided in the S1 File. The scale bar indicates the distance.

The various pairs of aligned amino acid sequences of the Vlp proteins differed at ≥10% of positions within a sub-family and ≥30% of positions between proteins of different sub-families. The diversity among the four identified *vsp* genes of *B*. *miyamotoi* LB-2001 was examined in more depth by adding to a codon-based nucleotide alignment the following: 3 *vsp* sequences of strain FR64b of *B*. *miyamotoi*, 4 *vsp* sequences of strains HS1 and YBT of *B*. *hermsii*, and 2 *vsp* sequences of strain Oz1 of *B*. *turicatae* (S1 File). The 3 *vsp* sequences of strain FR64b were the only ones identified by BLASTN searches with each of the 4 *vsp* LB-2001 sequences of all strain FR64b contigs deposited with GenBank. Fig 3 is an unrooted phylogram that shows diversity of the *B*. *miyamotoi vsp* genes, both within and between strains, that is as great as for two species, *B*. *hermsii* and *B*. *turicatae*, that have documented antigenic variation of Vsp proteins for immune evasion [29, 48]. The splits-decomposition analysis revealed a recombination network among the *B*. *miyamotoi vsp* genes that appears as deep as that previously demonstrated for *B*. *hermsii*’s *vsp* repertoire [49].

### Two loci with *vsp1* sequences

As noted, the lpB plasmid fragment bears the *vsp1* gene, but a 99.4% identical *vsp* sequence exists among a group of tandemly-arrayed *vsp* and *vlp* genes of lpD (Fig 1). Upstream of the two *vsp1* sequences are a BBB14-like pseudogene on lpB and a delta sub-family Vlp gene, *vlpD4*, on the lpD fragment. Downstream of both *vsp1* versions are sequences for delta sub-family Vlp proteins; these were designated *vlpD1* on lpB and *vlpD5* on lpD. There are at least 6 kb of *vsp* and *vlp* sequences 3’ to *vsp1* on lpB. The version of *vsp1* on lpD is followed over a length of 8 kb by 3 *vsp* and *vlp* sequences and beyond those by four other ORFs, including a homolog of the *B*. *burgdorferi* fibronectin-binding protein BBK32 (AAC66134).

**Fig 4:**
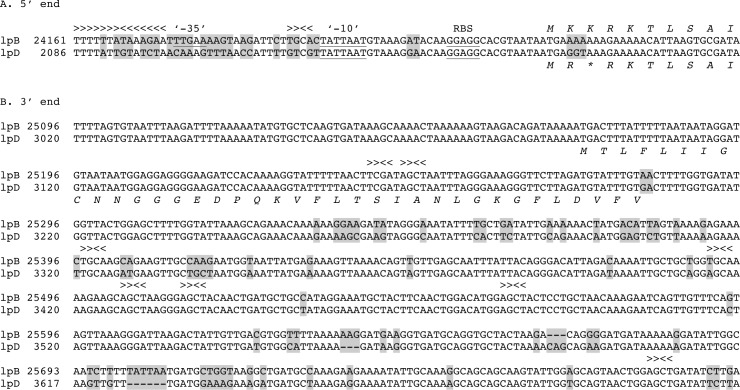
Alignments of nucleotide sequences of plasmid fragments lpB and lpD at the 5’ end of the respective *vsp1* gene sequences (section A) and downstream of the *vsp1* sequences in the region where the two sequences diverge again (section B). Between these 5’ and 3’ sequences the lpB and lpD were identical. The numbers on the left refer to the positions of accession numbers CP010328 for lpB and KR869o94 for lpD. Differences between the sequences are indicated by gray highlights. An inverted repeat in lpB but not lpD and the 4-base palindromes (TGCA/ACGT) occuring in either sequence are indicated by > and < marks. Consensus ribosome binding sequences and “-10” and “-35” elements of a RpoD-type promoter are indicated by underlining. In section A the translated amino acids for the starts of Vsp1 are shown for both sequences, either above or below the nucleotide sequence. In section B only the first 37 amino acids that was in common to both VlpD1 (lpB) and VlpD5 (lpD) are shown.

Fig 4 provides sequence-level comparisons of the 5’ and 3’ flanking regions for lpB and lpD. At the 5’ end (section A), both the lpB and lpD sequences have a consensus ribosomal binding sequences and what is compatible with a “-10” element of a RpoD-type promoter. Further upstream the sequences diverge, and notably there is a consensus RpoD “-35” element on the lpB sequence but not on the lpD sequence. The GGT trinucleotide of the initial coding sequence for the lpD *vsp1* replaces the AAA trinucleotide in the lpB *vsp1* and thereby creates a stop within 2 codons of the start. A stop codon at the beginning of some putative “silent” genes was also noted in our studies of *B*. *hermsii* and *B*. *turicatae* [25, 50, 51]. These substitutions may serve to further limit adventitious expression of *vsp* and *vlp* genes at loci other than a preferred expression site.

The lpD sequence also lacks the inverted repeat just in front of the putative “-35” element. A similarly-positioned inverted repeat before the promoter for the *ospC* gene on the cp26 plasmid of *B*. *burgdorferi* serves as the gene’s operator [52, 53]. OspC is homologous to the Vsp proteins [54]. The first ORF of the 6 kb cpD plasmid (KT355574) of *B*. *miyamotoi* is homologous to *B*. *burgdorferi*’s BBD18 protein, which is the repressor for the *ospC* operator [53]. Plausibly, this *B*. *miyamotoi* protein has a similar function in that species.

Section B of Fig 4 shows the post-*vsp1* region of each locus where the sequences diverge again downstream. This occurs after the codon for amino acid residue 37 of the following VlpD. Thereafter the deduced VlpD1 and VlpD5 proteins differed at 34 (11%) of 315 positions. The sequences in common between the two loci, even after they begin to differ, are notable for the frequency of TCGA/AGCT palindromes.

### RNA-seq

**Fig 5:**
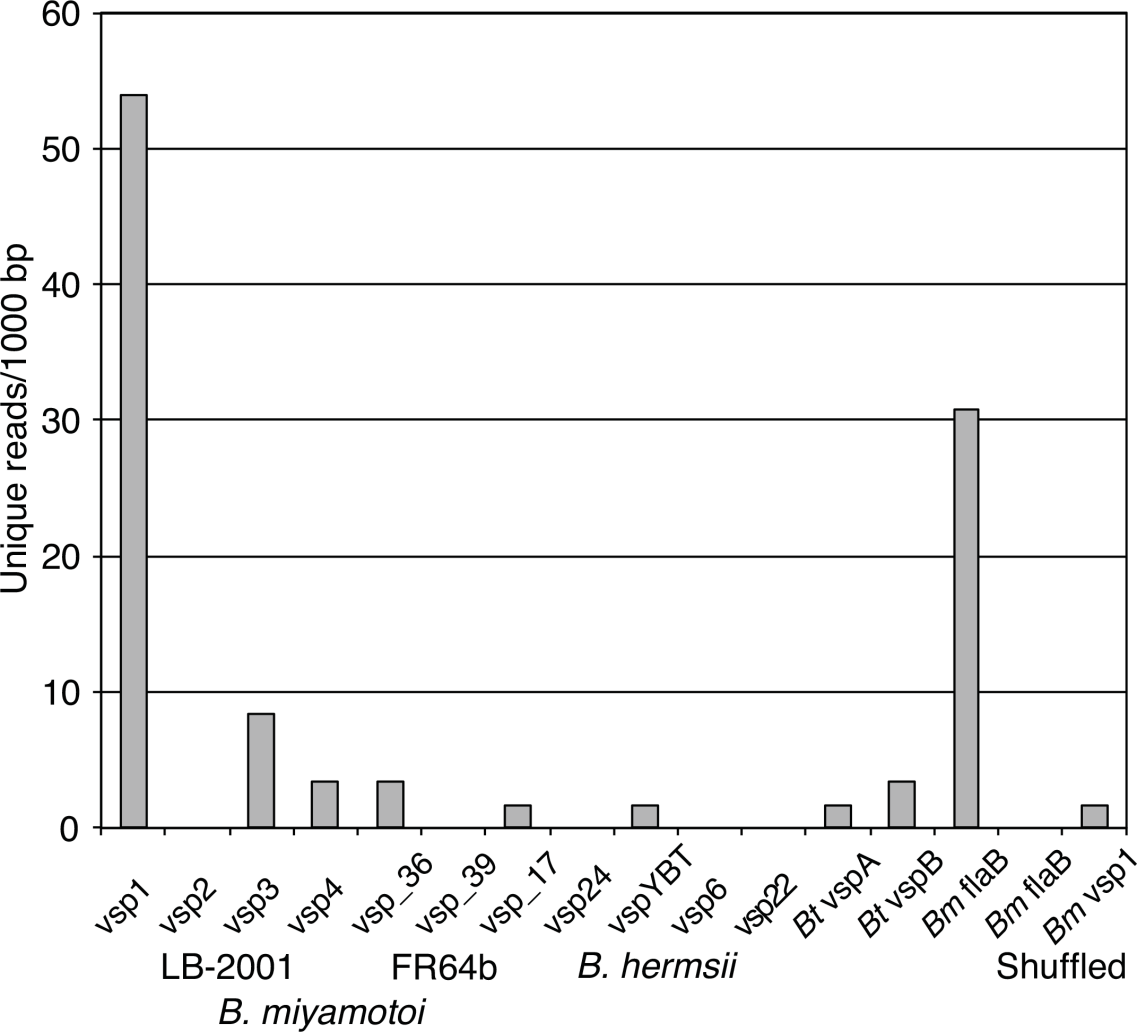
Histogram of numbers of unique RNA-seq reads mapping to different *vsp* sequences of strains LB-2001 and FR64b of *B*. *miyamotoi*, strains HS1 and YBT of *B*. *hermsii*, and strain Oz1 of *B*. *turicatae*, as well as the *flaB* sequence of *B*. *miyamotoi*. The sequences are the same as for Fig 3 and are provided in the S1 File. Shuffled versions of the latter sequence and the LB-2001 *vsp1* sequence were also included in the set.

Of 2,763,166 total reads of 50–200 nt and trimmed of low quality regions, 2,386 (0.1%) mapped to the 907,293 bp of LB-2001 chromosome (CP006647) or plasmid fragments lpB, lpD, and lpE, and 2,557,069 (92.5%) mapped to chromosomes 1–19, chromosomes X and Y, and the mitochondrion of *Mus musculus*. The mean number of the reads that uniquely mapped were 7.1 per 1000 bp for the *B*. *miyamotoi* sequences overall and 0.8 per 1000 bp of mouse genome, excluding the mitochondrion sequence. Though the number of *B*. *miyamotoi*-mapped reads was low, there appeared to be a sufficient quantity to evaluate expression of *vsp1* without resort to further PCR amplification with the risk of unrepresentative amplification. This was done by using as the target set for the analysis the 4 *vsp* genes for strain LB-2001, 3 *vsp* genes for strain FR64b, 4 *vsp* genes for *B*. *hermsii*, and 2 *vsp* genes for *B*. *turicatae* that were used for the phylogenetic analysis shown in Fig 3. The sequence of the *flaB* gene for the major component of the spirochete flagella served as a positive control, as it was anticipated to be one of the more highly expressed genes in vivo [55, 56]. As additional negative controls to the *vsp* sequences from *B*. *hermsii* and *B*. *turicatae*, the LB-2001 *vsp1* and *flaB* sequences were randomly shuffled. Fig 5 shows the length-adjusted values for reads uniquely mapping to the various sequences. Among the 4 known *vsp* sequences for LB-2001, *vsp1* stood out from *vsp2*, *vsp3*, and *vsp4* in the number of reads mapping to its sequence. This value exceeded that of reads mapping to the *flaB* sequence. A Poisson distribution described the counts of mapped reads by individual gene among the great excess of total reads. Under a null hypothesis of statistically indistinguishable frequencies of reads mapping to the different *vsp* sequences, the observed number for *vsp1* was highly improbable (*p* <0.0001). This was evidence but not proof that *vsp1* of lpB was the active *vsp* allele in the majority of the spirochetes growing in the blood of the mice.

## Conclusions

Did this study establish that antigen variation occurs during *B*. *miyamotoi* infection? No. This will likely require the demonstration of evasion of adaptive immunity by a switch of surface proteins during an experimental infection with a minimum infectious inoculum of a clonal population and then attribution of that switch to a reversible DNA rearrangement [57]. But the finding of full- or near-full length sequences for a minimum of 19 diverse *vsp* and *vlp* indicates that *B*. *miyamotoi* has the genetic building blocks for the type multi-phasic antigenic variation that characterizes relapsing fever [22]. In addition, the presence of two copies of a *vsp* gene, with one copy next to a candidate promoter, is consistent with a mechanism for antigenic variation that has been demonstrated in other species in the relapsing fever group. The identification of a putative expression site and the various *vsp* and *vlp* sequences provides the database for design of primers and nucleotide and protein microarrays for further studies of expression in the organism during infection and the immune responses of infected hosts.

The diversity of the partition proteins, like the ParA/PF32 homologs, indicates that there at least four different types of plasmids in *B*. *miyamotoi*. Are these the complete sequences of the plasmids represented here? This is undoubtedly not the case for the lpE fragment, which lacks the aformentioned plasmid replication and partition ORFs. And it is probably not the case for lpD, which encodes unique PF32, PF49, and PF50 proteins, but is shorter in length than the complete plasmids that bear *vsp* and *vlp* genes in *B*. *hermsii* and *B*. *turicatae* [27, 58]. The lpB sequence may be close to complete; it is similar in size to a *vsp*-bearing plasmid in strain FR64b [30]. But confirmation of that plasmid’s structure—and what the ends are, if linear [42]—require further study. While a circular topology for the lpB plasmid cannot be excluded at this point, the absence of telomeres could be incompatible with a mechanism of antigenic variation dependent on duplicative translocations between linear plasmids [27, 59].

## Supporting Information

S1 File NEXUS format codon-based alignment of partial nucleotide sequences of *vsp* genes of selected *Borrelia* species as described in legends for Figs 3 and 5. (NXS)

## References

[1] Krause PJ, Fish D, Narasimhan S, Barbour AG. *Borrelia miyamotoi* infection in nature and in humans. Clin Microbiol Infect. 2015;21: 631–639. 10.1016/j.cmi.2015.02.006 25700888 PMC4470780

[2] Wagemakers A, Staarink PJ, Sprong H, Hovius JW. *Borrelia miyamotoi*: a widespread tick-borne relapsing fever spirochete. Trends Parasitol. 2015;31: 260–269. 10.1016/j.pt.2015.03.008 25892254

[3] Barbour AG, Bunikis J, Travinsky B, Hoen AG, Diuk-Wasser MA, Fish D, et al Niche partitioning of *Borrelia burgdorferi* and *Borrelia miyamotoi* in the same tick vector and mammalian reservoir species. Am J Trop Med Hyg. 2009;81: 1120–1131. 10.4269/ajtmh.2009.09-0208 19996447 PMC2841027

[4] Padgett K, Bonilla D, Kjemtrup A, Vilcins IM, Yoshimizu MH, Hui L, et al Large scale spatial risk and comparative prevalence of *Borrelia miyamotoi* and *Borrelia burgdorferi* sensu lato in *Ixodes pacificus*. PLoS One. 2014;9(10): e110853 10.1371/journal.pone.0110853 25333277 PMC4205013

[5] Salkeld DJ, Cinkovich S, Nieto NC. Tick-borne pathogens in northwestern California, USA. Emerg Infect Dis. 2014;20: 493–494. 10.3201/eid2003.130668 24565119 PMC3944864

[6] Margos G, Stockmeier S, Hizo-Teufel C, Hepner S, Fish D, Dautel H, et al Long-term in vitro cultivation of *Borrelia miyamotoi* Ticks Tick Borne Dis. 2015;6: 181–184. 10.1016/j.ttbdis.2014.12.001 25561082

[7] Wagemakers A, Oei A, Fikrig MM, Miellet WR, Hovius JW. The relapsing fever spirochete *Borrelia miyamotoi* is cultivable in a modified Kelly-Pettenkofer medium, and is resistant to human complement. Parasit Vectors. 2014;7: 418 10.1186/1756-3305-7-418 25189195 PMC4261524

[8] Norris SJ, Howell JK, Garza SA, Ferdows MS, Barbour AG. High- and low-infectivity phenotypes of clonal populations of in vitro- cultured *Borrelia burgdorferi*. Infect Immun. 1995;63: 2206–2212. 7768600 10.1128/iai.63.6.2206-2212.1995 PMC173287

[9] Fukunaga M, Takahashi Y, Tsuruta Y, Matsushita O, Ralph D, McClelland M, et al Genetic and phenotypic analysis of *Borrelia miyamotoi* sp. nov., isolated from the ixodid tick *Ixodes persulcatus*, the vector for Lyme disease in Japan. Int J Syst Bacteriol. 1995;45: 804–810. 7547303 10.1099/00207713-45-4-804

[10] Platonov AE, Karan LS, Kolyasnikova NM, Makhneva NA, Toporkova MG, Maleev VV, et al Humans infected with relapsing fever spirochete *Borrelia miyamotoi*, Russia. Emerg Infect Dis. 2011;17: 1816–1823. 10.3201/eid1710.101474 22000350 PMC3310649

[11] Krause PJ, Narasimhan S, Wormser GP, Rollend L, Fikrig E, Lepore T, et al Human *Borrelia miyamotoi* infection in the United States. N Engl J Med. 2013;368: 291–293. 10.1056/NEJMc1215469 PMC3934646 23323920

[12] Fonville M, Friesema IH, Hengeveld PD, Docters van Leeuwen A, Jahfari S, Harms MG, et al Human exposure to tickborne relapsing fever spirochete *Borrelia miyamotoi*, the Netherlands. Emerg Infect Dis. 2014;20: 1244–1245. 10.3201/eid2007.131525 24963562 PMC4073841

[13] Sato K, Takano A, Konnai S, Nakao M, Ito T, Koyama K, et al Human infections with *Borrelia miyamotoi*, Japan. Emerg Infect Dis. 2014;20: 1391–1393. 10.3201/eid2008.131761 25061761 PMC4111186

[14] Krause PJ, Narasimhan S, Wormser GP, Barbour AG, Platonov AE, Brancato J, et al *Borrelia miyamotoi* sensu lato seroreactivity and seroprevalence in the northeastern United States. Emerg Infect Dis. 2014;20: 1183–1190. 10.3201/eid2007.131587 24960072 PMC4073859

[15] Molloy PJ, Telford SR, Chowdri HR, Lepore TJ, Gugliotta JL, Weeks KE, et al *Borrelia miyamotoi* disease (BMD) in the northeastern United States: a case series. Ann Int Med. 2015;163: 91–98. 10.7326/M15-0333 26053877

[16] Gugliotta JL, Goethert HK, Berardi VP, Telford SR, 3rd. Meningoencephalitis from *Borrelia miyamotoi* in an immunocompromised patient. N Engl J Med. 2013;368: 240–245. 10.1056/NEJMoa1209039 23323900 PMC4018741

[17] Hovius JW, de Wever B, Sohne M, Brouwer MC, Coumou J, Wagemakers A, et al A case of meningoencephalitis by the relapsing fever spirochaete *Borrelia miyamotoi* in Europe. Lancet. 2013;382: 658 10.1016/S0140-6736(13)61644-X 23953389 PMC3987849

[18] Scoles GA, Papero M, Beati L, Fish D. A relapsing fever group spirochete transmitted by *Ixodes scapularis* ticks. Vector Borne Zoonotic Dis. 2001;1: 21–34. 12653133 10.1089/153036601750137624

[19] Barbour AG. Phylogeny of a relapsing fever *Borrelia* species transmitted by the hard tick *Ixodes scapularis*. Infect Genet Evol. 2014;27: 551–558. 10.1016/j.meegid.2014.04.022 24813576 PMC4182126

[20] Sonenshine DE, Roe RM. Biology of ticks 2nd ed. New York: Oxford University Press; 2014.

[21] Barbour A. Relapsing fever In: Dennis DT, Goodman JL, Sonenshine DE, editors. Tick-borne diseases of humans. Washington, D.C.: ASM Press; 2005 p. 220–236.

[22] Barbour AG, Guo BP. The pathogenesis of relapsing fever In: Radolf JD, Samuels DS, editors. Borrelia: molecular biology, host interaction and pathogenesis. Norfolk, UK: Calister Academic Press; 2010 p. 333–358.

[23] Barbour AG, Dai Q, Restrepo BI, Stoenner HG, Frank SA. Pathogen escape from host immunity by a genome program for antigenic variation. Proc Natl Acad Sci U S A. 2006;103: 18290–18295. 17101971 10.1073/pnas.0605302103 PMC1635980

[24] Hinnebusch BJ, Barbour AG, Restrepo BI, Schwan TG. Population structure of the relapsing fever spirochete *Borrelia hermsii* as indicated by polymorphism of two multigene families that encode immunogenic outer surface lipoproteins. Infect Immun. 1998;66: 432–440. 9453591 10.1128/iai.66.2.432-440.1998 PMC107923

[25] Dai Q, Restrepo BI, Porcella SF, Raffel SJ, Schwan TG, Barbour AG. Antigenic variation by *Borrelia hermsii* occurs through recombination between extragenic repetitive elements on linear plasmids. Mol Microbiol. 2006;60: 1329–1343. 16796672 10.1111/j.1365-2958.2006.05177.x PMC5614446

[26] Meier JT, Simon MI, Barbour AG. Antigenic variation is associated with DNA rearrangements in a relapsing fever Borrelia. Cell. 1985;41: 403–409. 2580643 10.1016/s0092-8674(85)80013-1

[27] Pennington PM, Cadavid D, Bunikis J, Norris SJ, Barbour AG. Extensive interplasmidic duplications change the virulence phenotype of the relapsing fever agent *Borrelia turicatae*. Mol Microbiol. 1999;34: 1120–1132. 10594835 10.1046/j.1365-2958.1999.01675.x

[28] Raffel SJ, Battisti JM, Fischer RJ, Schwan TG. Inactivation of genes for antigenic variation in the relapsing fever spirochete *Borrelia hermsii* reduces infectivity in mice and transmission by ticks. PLoS Pathog. 2014;10: e1004056 10.1371/journal.ppat.1004056 24699793 PMC3974855

[29] Restrepo BI, Kitten T, Carter CJ, Infante D, Barbour AG. Subtelomeric expression regions of Borrelia hermsii linear plasmids are highly polymorphic. Mol Microbiol. 1992;6: 3299–3311. 1484486 10.1111/j.1365-2958.1992.tb02198.x

[30] Hamase A, Takahashi Y, Nohgi K, Fukunaga M. Homology of variable major protein genes between *Borrelia hermsii* and *Borrelia miyamotoi*. FEMS Microbiol Lett. 1996;140: 131–137. 8764474 10.1016/0378-1097(96)00168-1

[31] Eid J, Fehr A, Gray J, Luong K, Lyle J, Otto G, et al Real-Time DNA sequencing from single polymerase molecules. Science. 2009;323: 133–138. 10.1126/science.1162986 19023044

[32] Hue F, Ghalyanchi Langeroudi A, Barbour AG. Chromosome sequence of *Borrelia miyamotoi*, an uncultivable tick-borne agent of human infection. Genome Announc. 2013;1 e00713–13. 10.1128/genomeA.00713-13 24029760 PMC3772144

[33] Myers EW, Sutton GG, Delcher AL, Dew IM, Fasulo DP, Flanigan MJ, et al A whole-genome assembly of Drosophila. Science. 2000;287: 2196–2204. 10731133 10.1126/science.287.5461.2196

[34] Altschul SF, Madden TL, Schaffer AA, Zhang J, Zhang Z, Miller W, et al Gapped BLAST and PSI-BLAST: a new generation of protein database search programs. Nucleic Acids Res. 1997;25: 3389–3402. 9254694 10.1093/nar/25.17.3389 PMC146917

[35] Casjens S, Palmer N, van Vugt R, Huang WM, Stevenson B, Rosa P, et al A bacterial genome in flux: the twelve linear and nine circular extrachromosomal DNAs in an infectious isolate of the Lyme disease spirochete *Borrelia burgdorferi*. Mol Microbiol. 2000;35: 490–516. 10672174 10.1046/j.1365-2958.2000.01698.x

[36] Fraser CM, Casjens S, Huang WM, Sutton GG, Clayton R, Lathigra R, et al Genomic sequence of a Lyme disease spirochaete, *Borrelia burgdorferi*. Nature. 1997;390: 580–586. 9403685 10.1038/37551

[37] Larkin MA, Blackshields G, Brown NP, Chenna R, McGettigan PA, McWilliam H, et al Clustal W and Clustal X version 2.0. Bioinformatics. 2007;23: 2947–2948. 17846036 10.1093/bioinformatics/btm404

[38] Gouy M, Guindon S, Gascuel O. SeaView version 4: A multiplatform graphical user interface for sequence alignment and phylogenetic tree building. Mol Biol Evol. 2010;27: 221–224. 10.1093/molbev/msp259 19854763

[39] Huson DH, Bryant D. Application of phylogenetic networks in evolutionary studies. Mol Biol Evol. 2006;23: 254–267. 16221896 10.1093/molbev/msj030

[40] Zhong J, Skouloubris S, Dai Q, Myllykallio H, Barbour AG. Function and evolution of plasmid-borne genes for pyrimidine biosynthesis in *Borrelia* spp. J Bacteriol. 2006;188: 909–918. 16428394 10.1128/JB.188.3.909-918.2006 PMC1347342

[41] Lescot M, Audic S, Robert C, Nguyen TT, Blanc G, Cutler SJ, et al The genome of *Borrelia recurrentis*, the agent of deadly louse-borne relapsing fever, is a degraded subset of tick-borne *Borrelia duttonii*. PLoS Genet. 2008;4: e1000185 10.1371/journal.pgen.1000185 18787695 PMC2525819

[42] Miller SC, Porcella SF, Raffel SJ, Schwan TG, Barbour AG. Large linear plasmids of *Borrelia* species that cause relapsing fever. J Bacteriol. 2013;195: 3629–3639. 10.1128/JB.00347-13 23749977 PMC3754566

[43] Jewett MW, Lawrence K, Bestor AC, Tilly K, Grimm D, Shaw P, et al The critical role of the linear plasmid lp36 in the infectious cycle of *Borrelia burgdorferi*. Mol Microbiol. 2007;64: 1358–7134. 17542926 10.1111/j.1365-2958.2007.05746.x PMC1974800

[44] Margolis N, Hogan D, Tilly K, Rosa PA. Plasmid location of *Borrelia* purine biosynthesis gene homologs. J Bacteriol. 1994;176: 6427–32. 7961392 10.1128/jb.176.21.6427-6432.1994 PMC196994

[45] Kobryn K, Chaconas G. Hairpin telomere resolvases. Microbiol Spectr. 2014;2 10.1128/microbiolspec.MDNA3-0023-2014 26104454

[46] Barbour AG, Carter CJ, Sohaskey CD. Surface protein variation by expression site switching in the relapsing fever agent *Borrelia hermsii*. Infect Immun. 2000;68: 7114–7121. 11083837 10.1128/iai.68.12.7114-7121.2000 PMC97822

[47] Chaconas G. Hairpin telomeres and genome plasticity in *Borrelia*: all mixed up in the end. Mol Microbiol. 2005;58: 625–35. 16238614 10.1111/j.1365-2958.2005.04872.x

[48] Cadavid D, Thomas DD, Crawley R, Barbour AG. Variability of a bacterial surface protein and disease expression in a possible mouse model of systemic Lyme borreliosis. J Exp Med. 1994;179: 631–642. 8294872 10.1084/jem.179.2.631 PMC2191368

[49] Rich SM, Sawyer SA, Barbour AG. Antigen polymorphism in *Borrelia hermsii*, a clonal pathogenic bacterium. Proc Natl Acad Sci U S A. 2001;98: 15038–15043. 11742066 10.1073/pnas.071042098 PMC64979

[50] Cadavid D, Pennington PM, Kerentseva TA, Bergstrom S, Barbour AG. Immunologic and genetic analyses of VmpA of a neurotropic strain of *Borrelia turicatae*. Infect Immun. 1997;65: 3352–3360. 9234797 10.1128/iai.65.8.3352-3360.1997 PMC175474

[51] Pennington PM, Cadavid D, Barbour AG. Characterization of VspB of *Borrelia turicatae*, a major outer membrane protein expressed in blood and tissues of mice. Infect Immun. 1999;67: 4637–4645. 10456910 10.1128/iai.67.9.4637-4645.1999 PMC96788

[52] Xu Q, McShan K, Liang FT. Verification and dissection of the *ospC* operator by using *flaB* promoter as a reporter in *Borrelia burgdorferi*. Microb Pathog. 2008;45: 70–78. 10.1016/j.micpath.2008.03.002 18479884 PMC2497006

[53] Sarkar A, Hayes BM, Dulebohn DP, Rosa PA. Regulation of the virulence determinant OspC by bbd18 on linear plasmid lp17 of *Borrelia burgdorferi*. J Bacteriol. 2011;193: 5365–5373. 10.1128/JB.01496-10 21784941 PMC3187453

[54] Carter CJ, Bergstrom S, Norris SJ, Barbour AG. A family of surface-exposed proteins of 20 kilodaltons in the genus *Borrelia*. Infect Immun. 1994;62: 2792–2799. 8005669 10.1128/iai.62.7.2792-2799.1994 PMC302883

[55] Pennington PM, Allred CD, West CS, Alvarez R, Barbour AG. Arthritis severity and spirochete burden are determined by serotype in the *Borrelia turicatae*-mouse model of Lyme disease. Infect Immun. 1997;65: 285–292. 8975925 10.1128/iai.65.1.285-292.1997 PMC174589

[56] Zhong J, Barbour AG. Cross-species hybridization of a *Borrelia burgdorferi* DNA array reveals infection- and culture-associated genes of the unsequenced genome of the relapsing fever agent *Borrelia hermsii*. Mol Microbiol. 2004;51: 729–748. 14731275 10.1046/j.1365-2958.2003.03849.x

[57] Barbour AG, Restrepo BI. Antigenic variation in vector-borne pathogens. Emerg Infect Dis. 2000;6: 449–457. 10998374 10.3201/eid0605.000502 PMC2627965

[58] Plasterk RH, Simon MI, Barbour AG. Transposition of structural genes to an expression sequence on a linear plasmid causes antigenic variation in the bacterium *Borrelia hermsii*. Nature. 1985;318: 2572–63. 10.1038/318257a0 4069202

[59] Kitten T, Barbour AG. Juxtaposition of expressed variable antigen genes with a conserved telomere in the bacterium *Borrelia hermsii*. Proc Natl Acad Sci U S A. 1990;87: 6077–6081. 2385585 10.1073/pnas.87.16.6077 PMC54475

